# Case Report: Mechanical thrombectomy using stent retriever devices in deep cerebral venous thrombosis: illustrative cases

**DOI:** 10.3389/fstro.2025.1568841

**Published:** 2025-04-23

**Authors:** Álvaro Martínez-Martín, Francisco Hernández-Fernández, Juan David Molina-Nuevo, Blanca Serrano Serrano, Tomás Segura

**Affiliations:** ^1^Neurology Department, Complejo Hospitalario Universitario de Albacete, Albacete, Spain; ^2^Radiology Department, Complejo Hospitalario Universitario de Albacete, Albacete, Spain

**Keywords:** cerebral venous thrombosis, endovascular treatment, mechanical thrombectomy, stent retriever, contraceptives (oral)

## Abstract

**Background:**

Treatment of cerebral venous thrombosis has traditionally been based on anticoagulant therapy. However, in certain circumstances, such as deep cerebral venous thrombosis, anticoagulation may be insufficient, so endovascular treatment by mechanical thrombectomy has been used for some years. There is currently no clear indication of which device is the gold standard in the endovascular treatment of cerebral venous thrombosis, although stent retriever devices are the most commonly used.

**Case presentation:**

We describe two cases of deep cerebral venous thrombosis refractory to anticoagulant treatment treated by mechanical thrombectomy with stent retriever devices, one of which has not been described to date as being used in the treatment of cerebral venous thrombosis (Tiger XL^®^).

**Conclusions:**

Some situations in like deep cerebral venous thrombosis may require endovascular treatment with mechanical thrombectomy if anticoagulation fails, with increasing evidence that it improves vital and functional prognosis.

## Background

Cerebral venous thrombosis (CVT) is a rare cause of stroke, occurring especially in young adults and females (Silvis et al., 2017). This higher incidence in females is related to risk factors involved in its pathophysiology, such as pregnancy, puerperium and contraceptive use (Silvis et al., 2017). Its clinical presentation is broad, ranging from mild manifestations such as headache to the more severe such as coma (Stam, 2005). This diverse form of presentation makes it necessary to maintain a high level of diagnostic suspicion to avoid diagnostic delay, which can be deleterious for the patient, both in terms of morbidity and mortality (Lee et al., 2018). Involvement of the deep cerebral venous system (DCVT) increases morbidity and mortality in these patients, with an increased risk of both venous infarction and hemorrhagic transformation (Stolz, 2008). Diagnosis is based on neuroimaging using brain CT and/or MRI, with or without the use of specific venography sequences (Silvis et al., 2017; Stam, 2005; Lee et al., 2018). Use of transcranial Doppler (TCD) has been proposed as an alternative to diagnosis and even during the follow-up of CVT, as it allows the assessment of hemodynamic factors (unlike angioCT or angioMRI) that could have an implication on the functional prognosis of patients. However, currently it is not clear whether TCD can play a useful role in monitoring and steering anticoagulation treatment in CVT (Stolz, 2008).

The classical therapeutic approach to CVT is based on anticoagulation (Silvis et al., 2017; Stam, 2005; Lee et al., 2018), but there are some clinical situations in which this may not be sufficient, especially in the case of DCVT. Recently, endovascular treatment has become relevant in these cases, in particular mechanical thrombectomy (MT) (Silvis et al., 2017; Stam, 2005; Lee et al., 2018; Yeo et al., 2020; Coutinho et al., 2020). Anticoagulation therapy prevents thrombus growth or embolization, whereas MT allows rapid sinus recanalization (Coutinho et al., 2020). According to current clinical guidelines (Ferro et al., 2017), in patients presenting with altered mental status, coma, DCVT or intracranial hemorrhage, the risk of a fatal outcome is higher and endovascular treatment should be considered.

We present two cases of DCVT refractory to anticoagulation therapy which were subjected to MT as a rescue treatment.

## Case presentation

### Case 1

**Figure 1:**
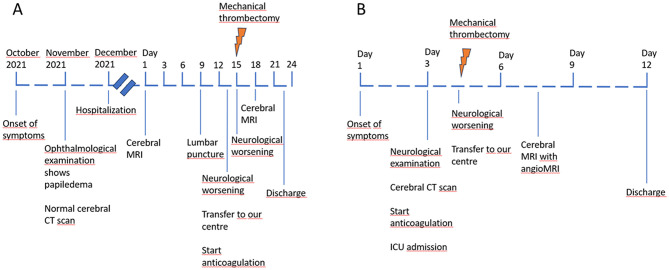
Timeline scheme for case 1 **(A)** and 2 **(B)**.

A 29-year-old woman with overweight and treatment with oral contraceptives (estrogen and gestagen) as a risk factors, seen in the emergency department because of headache, intracranial hypertension and diplopia. Physical examination revealed papilledema. A cerebral CT scan performed without contrast was normal. Lumbar puncture was carried out, with an opening pressure of 44 cmH_2_O (47 cmH_2_O after the Valsalva maneuver); cytobiochemistry of the cerebrospinal fluid was normal. The study was completed with cerebral MRI, which showed thrombosis of the sigmoid sinus and extension to the right transverse sinus (see Figure 1A for timeline scheme).

**Figure 2:**
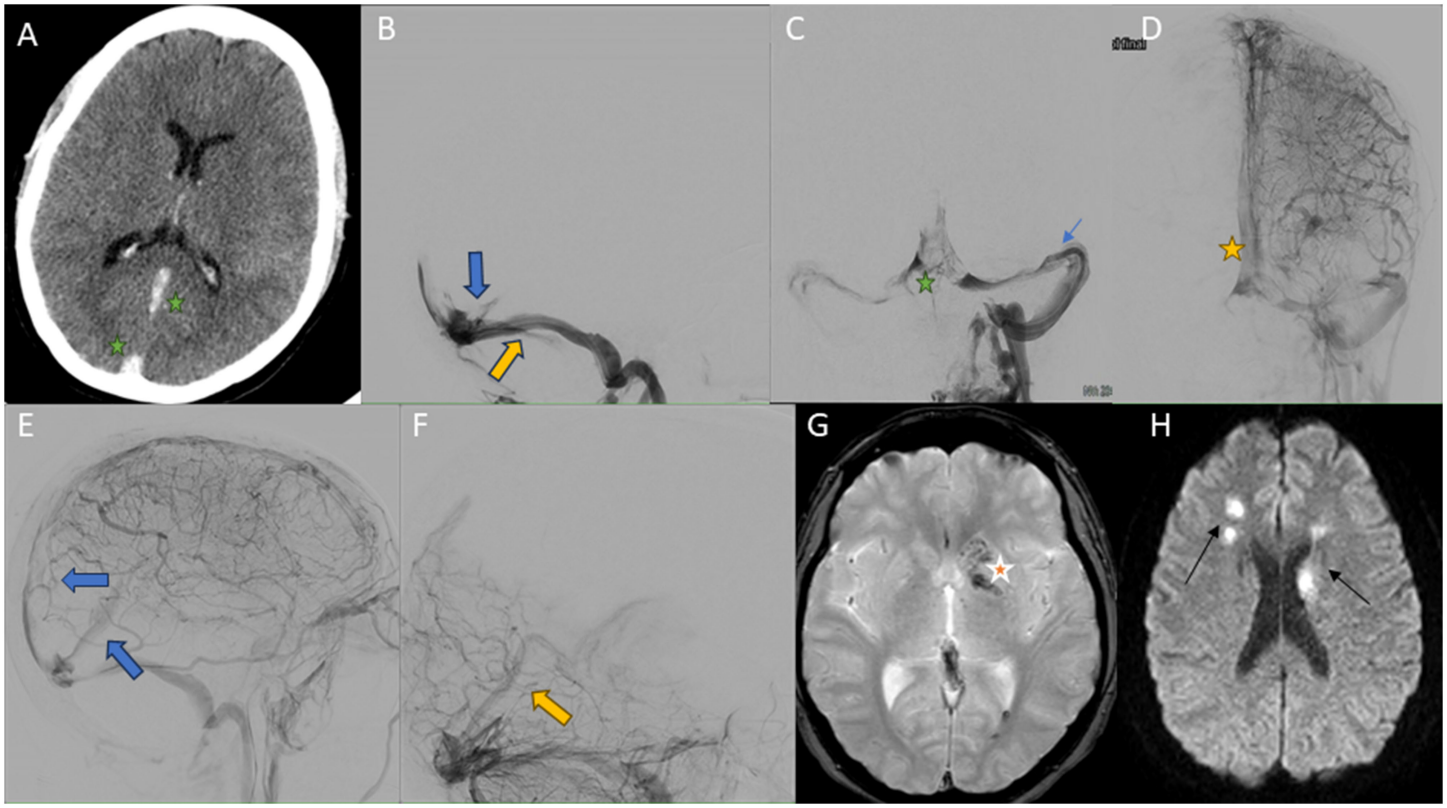
Images obtained during the course of case 1. **(A)** Cerebral CT scan without intravenous contrast. CVT with hyperdensity of superior sagittal sinuses (green star), rectus sinus (green star), vein of Galen, and internal cerebral veins. **(B)** Cerebral venography obtained by left internal jugular access, lateral view. An extensive thrombus with involvement of the rectus sinus is visualized (blue arrow). Direct aspiration was performed with the Neuron MAX 0.88^®^ device (yellow arrow), followed by access to the rectal sinus with a microcatheter and TigerXL^®^ device (not shown in the image). **(C)** Cerebral venography obtained by left internal jugular access, anteroposterior view. Recanalization of left transverse-sigmoid sinuses (green star) is evident after manual and pump aspiration through Neuron MAX 0.88^®^ and JET7^®^. Guide catheter is can be seen in these sinuses (blue arrow). **(D, E)** Selective left internal carotid arteriography cerebral arteriography in venous phase, anteroposterior **(D, E)** view. Repermeabilization of the previously occluded sinuses can be seen (superior sagittal sinus [yellow star in **(D)** and blue arrow in **(E)**], rectus sinus and deep cerebral veins [blue arrow in **(E)**]), with residual partial thrombosis persisting at the torcula. **(F)** Selective left vertebral arteriography lateral view. Recovery of deep venous drainage (yellow arrow). **(G, H)** Brain MRI, susceptibility-weighted **(G)** and diffusion weighted images **(H)**. Multiple signal alterations with diffusion restriction affecting bilateral basal ganglia, right thalamus and supratentorial white matter, compatible with venous infarcts (black arrows) with hemorrhagic transformation (red star).

The evolution was torpid, with worsening of the headache, mild right hemiparesis and encephalopathy. CT scan was repeated, showing signs of CVT (Figure 2A). She was transferred to our center, where anticoagulation with low molecular weight heparin at a dose of 1 mg/kg/12 h was started. After 12 h she presented with impaired consciousness, global aphasia and right hemiparesis. An urgent electroencephalogram was performed, which ruled out a critical or post-critical neurological deficit. The TCD study showed hyperpulsatility and high resistance of both middle cerebral arteries, suggestive of severe intracranial hypertension, for which treatment was started with postural measures and mannitol. A complete analytical study was performed, in which only positive ANAs with a 1/160 speckled pattern (without evidence of underlying rheumatological pathology), a folic acid deficiency (1.1 ng/mL) and mild hyperhomocysteinemia (15.3 μmol/L) were detected, with the rest of the thrombophilia study being normal.

The study was completed with cerebral arteriography, which showed large CVT with involvement of the sagittal and transverse sinuses and torcula, as well as involvement of the deep venous system (Figure 2B). MT was performed with manual and pump aspiration with Neuron MAX 0.88^®^ and JET7^®^ devices, as well as with a stent retriever (Trevo^®^ 6 mm). A 4F diagnostic catheter was placed in the vertebral artery for successive diagnostic series. Cerebral venous circulation was accessed by puncture of the left internal jugular vein, placing a guide catheter in the sigmoid sinus and the beginning of the transverse sinus. Successive aspirative MT were performed on the left transverse dural sinus, right transverse sinus, superior sagittal sinus and with stent retriever in the rectus sinus (total passes: 6), removing large amount of clot. Final controls showed recovery of the deep venous drainage (Figures 2C–F). At 24 h, the patient demonstrated complete neurological recovery. A control TCD study was performed, which showed reduced pulsatility and venous flow indices corresponding to deep venous system turbulence (vein of Rosenthal), which may it be suggestive of partial recanalization. Cerebral MRI was completed and revealed a patchy venous infarction with hemorrhagic transformation (Figures 2G, H). She was discharged with discontinuation of oral contraception, treatment with folic acid 5 mg/24 h and anticoagulation with acenocumarol (vitamin K inhibitor). The modified Rankin Scale was 0 at discharge and at 3 months.

### Case 2

**Figure 3:**
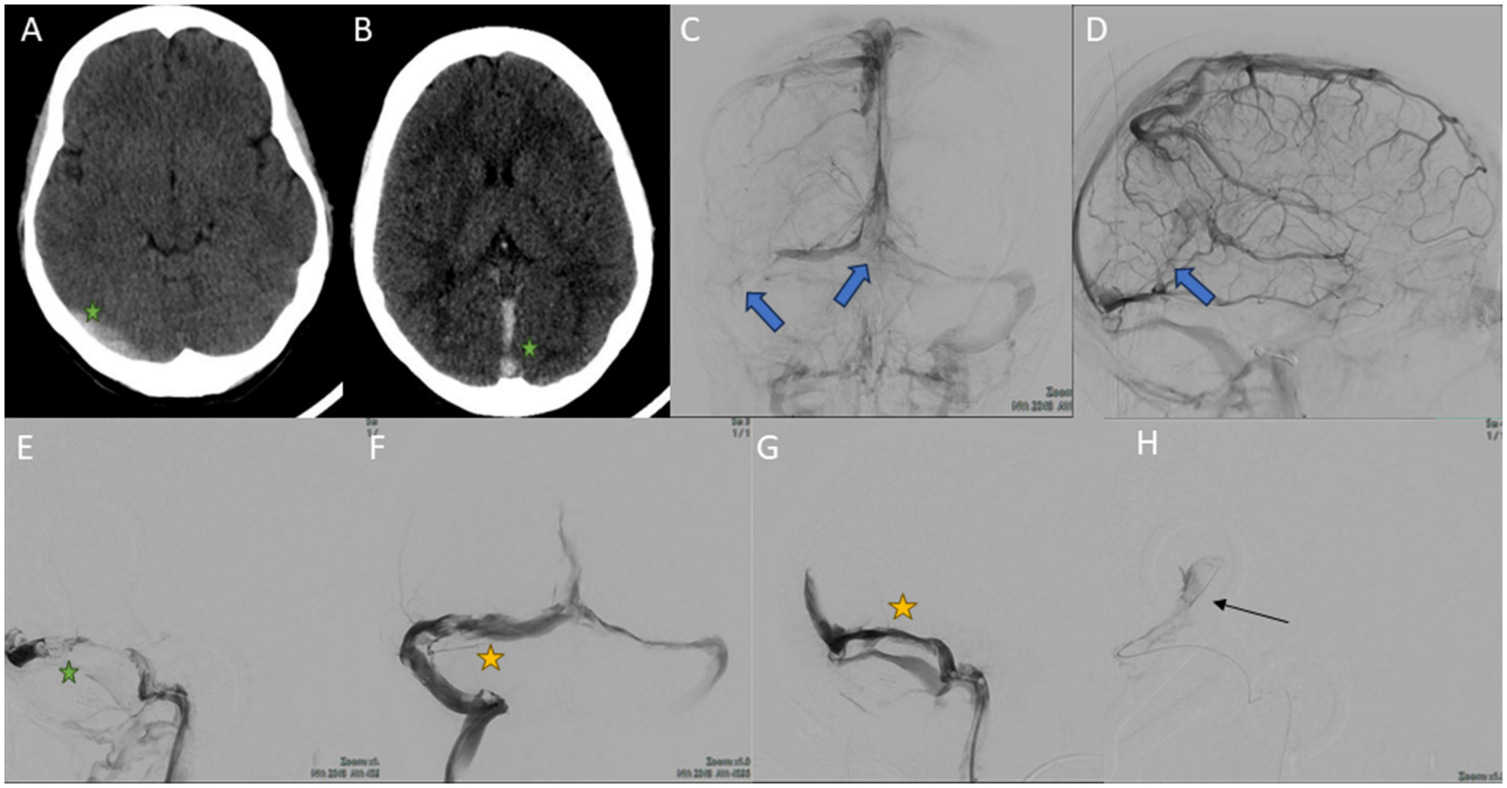
Images obtained during the course of case 2. **(A, B)** Cerebral CT scan without intravenous contrast. There is evidence of hyperdensity at the right transverse venous sinus, rectus sinus and superior sagittal sinus (green stars) due to acute thrombosis. **(C, D)** Selective right internal carotid arteriography in venous phase, anteroposterior **(C)** and lateral **(D)** view. Multiple repletion defects are evident in both transverse sinuses [blue arrow in **(C)**], torcula [blue arrow in **(C)**], superior sagittal sinus, rectus sinus and deep cerebral venous system [blue arrow in **(D)**], secondary to thrombosis. **(E–H)** Cerebral venography obtained by left internal jugular access, MT. **(E)** Lateral view. Occlusion of the right transverse sinus (green star). **(F)** Anteroposterior view. Recanalization of the right transverse sinus after endovascular treatment (yellow star). **(G)** Lateral view. Occlusion of the rectus sinus is seen (yellow star). **(H)** Lateral image view. Microcatheter (black arrow) is advanced through the rectus sinus prior to recanalization by stent retriever with TigerXL^®^ device (not shown in the image).

A 25-year-old woman with polycystic ovary syndrome on treatment with an intravaginal contraceptive as a risk factors was seen in the emergency department for progressive headache, encephalopathy and generalized weakness. Physical examination revealed peripapillary hemorrhages in the ocular fundus, with no associated neurological deficits (see Figure 1B for timeline scheme). A non-contrast cranial CT scan was performed, which showed evidence of CVT (Figures 3A, B). Anticoagulation with intravenous sodium heparin was started. The patient's level of consciousness suddenly deteriorated after 24 h and she was transferred to our center.

**Figure 4:**
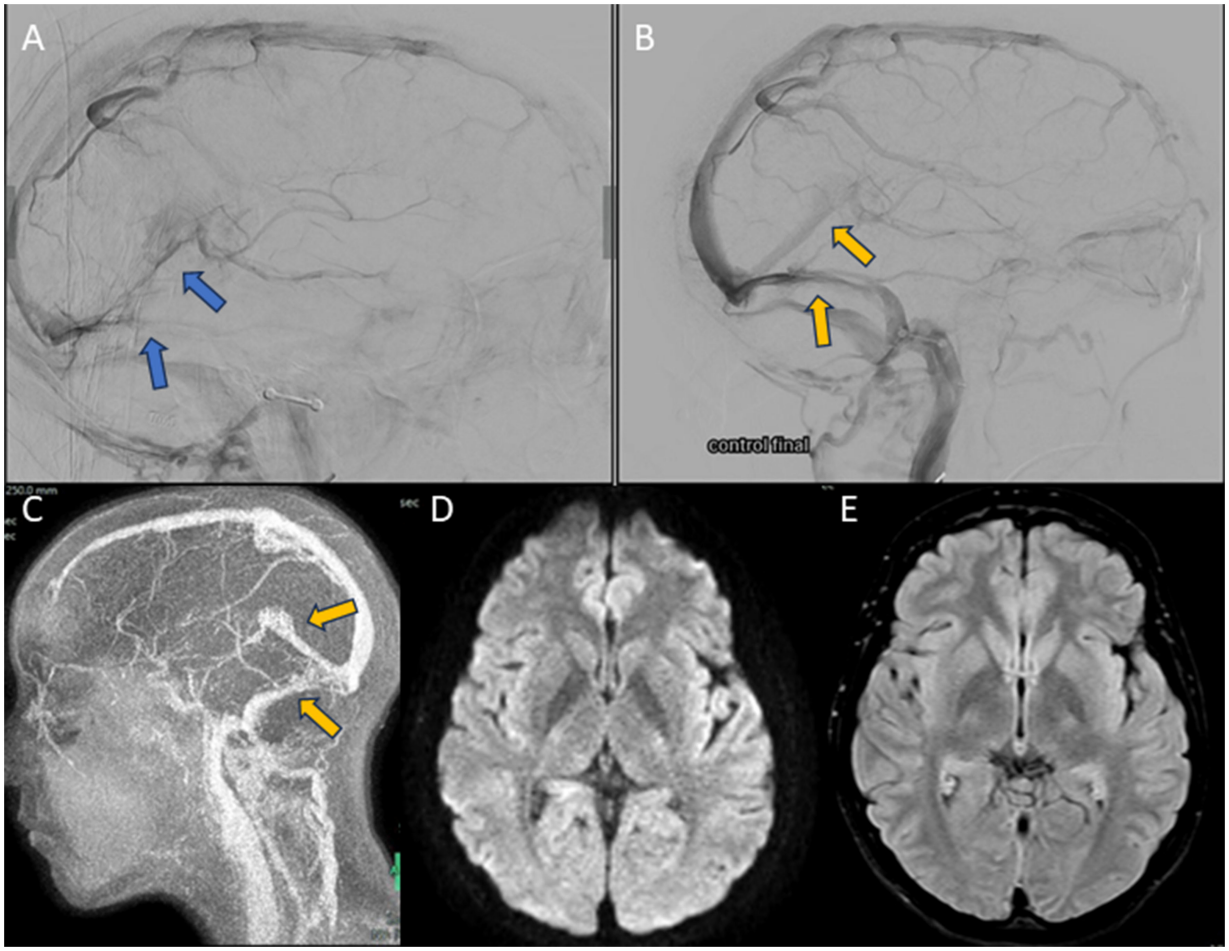
Images obtained during the course of case 2. **(A, B)** Selective right internal carotid arteriography in venous phase, lateral views. **(A)** Pre-MT image. Thrombosis of rectus sinus, deep cerebral veins, transverse and sigmoid sinuses (blue arrows) is seen. **(B)** Post-MT image. Recanalization of the rectus sinus (yellow arrow), torcula, superior sagittal sinus, and both transverse sinuses (yellow arrow) is seen. **(C–E)** Brain MRI performed 7 days after MT. **(C)** 2D-TOF MRI angiography, sagittal view. Shows the complete recovery of the venous system (yellow arrow). **(D)** DWI sequence. No acute ischaemic lesions are evident. **(E)** FLAIR sequence. No images suggestive of residual ischaemic or haemorrhagic pathology are seen.

Urgent cerebral arteriography was performed, which showed evidence of large CVT, again with DCVT (Figures 3C, D). MT was performed with Neuron MAX 0.88^®^ and JET7^®^ devices, as well as with a stent retriever (Tiger XL^®^) (Figures 3E–H). A 4F diagnostic catheter was placed in the right internal carotid artery for successive diagnostic series. Cerebral venous circulation was accessed by puncture of the right internal jugular vein by placing a guide catheter into the sigmoid sinus and the beginning of the transverse sinus. Successive aspirative MT were performed on the left transverse dural sinus, right transverse sinus, superior sagittal sinus, and with stent retriever in the rectus sinus (total passes: 6), removing again a large amount of clot. Final controls showed complete recanalization of the dural sinuses (Figures 4A, B). Cranial CT scan performed 24 h after MT showed no ischaemic or hemorrhagic complications. Neurological recovery was complete in the following days, with papilledema predominantly in the right eye and headache that improved with simple analgesia. As part of the etiological study, a complete blood analysis was performed, which only showed hypohomocysteinemia (2.1 μmol/L), the remainder being normal (including the thrombophilia study). The study was completed with cerebral MRI with angioMRI at 7 days, with no evidence of venous infarction and nearly complete recanalization of the venous system (Figures 4C–E). At discharge, intravaginal contraception was suspended and treatment was started with folic acid 5 mg/24 h and anticoagulation with acenocoumarol (vitamin K inhibitor). The modified Rankin Scale was 0 at discharge at 3 months.

## Discussion and conclusions

Classically, the gold standard treatment of CVT has been anticoagulation [preferably with low molecular weight heparins vs. unfractionated (Lee et al., 2018)] for 3–12 months (Silvis et al., 2017), regardless of the existence of associated intracranial hemorrhages, and this is still the recommendation of the current guidelines (Ferro et al., 2017). However, there are certain predictors of mortality in these patients in whom anticoagulation may not be sufficient (Siddiqui et al., 2015): rapid neurological deterioration despite anticoagulation, coma, intracranial hemorrhage, DCVT and posterior fossa involvement. In these patients, early endovascular treatment could be an adjuvant to anticoagulation, either by thrombolysis or by MT. Thrombolytic therapy [first applied in 1988 (Lee et al., 2018)] seems to have shown more limited efficacy in cases with high thrombus burden or extensive involvement of multiple venous sinuses, with MT allowing potentially faster recanalization (Siddiqui et al., 2015).

In recent years, there has been growing scientific evidence for the use of MT in the treatment of CVT, especially in the above-mentioned scenarios. Multiple techniques have been used (Lee et al., 2018): direct aspiration, use of stent retrievers, balloon-based MT, balloon and stent angioplasty, as well as different devices [AngioJetTM, PenumbraTM, MERCI retriever^®^, etc. (Siddiqui et al., 2015)]. To date, the largest clinical trial to assess the efficacy of endovascular treatment (both thrombolysis and MT) compared to medical treatment has been the TO-ACT trial (Coutinho et al., 2020). As authors discuss, because of the open-label design, physician choices regarding medical management after the acute phase could have influenced the outcome of patients. The small sample size, as well as the fact that some currently available devices were under development at the time of the trial (as low use of stent retriever devices show, only 15%), may explain why it was stopped early, in part due to futility. The endovascular treatment strategy was largely at the discretion of the interventionalist, so heterogeneity of the treatments could explain that no significant differences were found with the medical treatment. Another limitation were patient selection based on clinical previously reported presentations suspected to negatively influence outcomes. Therefore, subgroups that might actually show considerable benefits remained undetected, and there were not non-significant but clinically relevant treatment effects found (Bücke et al., 2022). Despite this, recanalization rates reported by different studies reach 70%−90% (Silvis et al., 2017).

To date, the optimal endovascular treatment in patients with CVT is not defined, and there are results for multiple devices used, with variable efficacies (Table 1; Quealy, 2024): stent retriever (Peng et al., 2022), stent retriever with direct aspiration (Lau et al., 2020), stent retriever with local thrombolysis (Wang et al., 2020), aspiration devices (Yeo et al., 2020), AngioJetTM (Siddiqui et al., 2015), balloon anchor with mobile aspiration (Matsuda et al., 2018), etc. Several systematic reviews have been conducted on the use of MT in the treatment of CVT and its comparison with medical treatment (Yeo et al., 2020; Siddiqui et al., 2015; Ilyas et al., 2017). The results seen in all of these studies, in general [and similar to the TO-ACT trial (Coutinho et al., 2020)], conclude that medical treatment achieves better results in terms of functional independence and mortality, although this is a biased result: those patients who are referred to MT are those with a more severe clinical presentation at onset and greater thrombotic burden, with failure of anticoagulant treatment and rescued by MT (Yeo et al., 2020), which inherently confers a worse prognosis (Ilyas et al., 2017). Therefore, in cases refractory to anticoagulation therapy, with contraindications to anticoagulation (Ilyas et al., 2017), thrombus length >10 cm, superior sagittal sinus involvement (Peng et al., 2022) or pre-treatment intracerebral hemorrhage (Yeo et al., 2020), MT would be an appropriate treatment.

**Table 1:** Efficacy of different endovascular treatments in CVT.

**Technique**	**Rate of complete recanalization**
Thrombo-aspiration	25.3%
Balloon thrombectomy	51.2%
Balloon angioplasty	33.9%
Stent angioplasty	36.4%
Stent retriever	37%
Microsnare	0%
AngioJet TM	55%

Modified from Quealy (2024).

Regarding the use of endovascular devices, the current trend is to use stent retriever devices (Siddiqui et al., 2015; Bücke et al., 2022; Quealy, 2024; Peng et al., 2022; Lau et al., 2020; Wang et al., 2020) as they can be used alone or as an anchor for a distal aspiration device (Lee et al., 2018) or in conjunction with local thrombolytic therapy (Wang et al., 2020). In our case, we report two cases of MT for the treatment of CVT using stent retriever devices, one of which is the novel Tiger XL^®^, unreported to date for use in the treatment of venous thrombosis. Its adjustable diameter reduces resistance to thrombus removal and the continuous braiding reduces the risk of thrombus detachment during removal. Its length is 53 mm and its size is from 1.5–9 mm; we expanded it slow and progressively to its full size (9 mm). Accordingly with the Triger Trial (Gupta et al., 2021) the use of Tigertriever devices in large vessel occlusions shows reduced procedure times compared with other stentriever devices as Solitaire^®^ or Trevo^®^. This potentially higher efficacy of Tigertriever devices might be caused by the technical specifications such as its manual adjustability of the radial force and with the possibility to interact with the clot in combination with the larger size of the Tiger XL^®^ (Maus et al., 2021), aspects that may be essential for treatment in a particular anatomy such as that of the cerebral venous sinuses. Compared with other static stentriever devices as Solitaire^®^ or Trevo^®^, relaxing the Tiger XL^®^ upon removal may also reduce the risk of vessel distortion, injury, and hemorrhagic complications. This can be particularly important in distal locations on arterial vessels and in cerebral venous sinuses. These special features offer greater user control regardless of anatomy or clot composition (Jankowitz et al., 2024). Trigertriever devices were not available during TO-ACT trial (Coutinho et al., 2020), so its efficacy could not been demonstrated during the trial. The functional outcome in our patients was excellent, with complete neurological recovery (modified Rankin Scale 0 at discharge) and virtually complete recanalization rates, indicating the safety and efficacy of these stent retriever devices. More in detail, accordingly with the Tiger Trial (Gupta et al., 2021) the primary safety composite endpoint rate of mortality and symptomatic intracranial hemorrhage was 18.1% (comparable to the 20.4% historical rate). A very low symptomatic intracranial hemorrhage rate was observed (1.7%). However, randomized clinical trials comparing the efficacy and safety of the different stentrievers are needed to establish whether there is any safety concerns specific to Tiger XL^®^ compared to other devices.

The main strength of this study is that it offers a therapeutic alternative to a potentially serious disease that significantly improves the functional prognosis of patients. The increased availability of intravascular devices today [compared to TO-ACT trial (Coutinho et al., 2020)] allows for adaptation to the complexity of different cases of CVT, which may improve the prognosis for functional recovery of patients. This statement, however, needs to be confirmed by further randomized clinical trials; a subgroup analysis of the efficacy of the different intravascular devices used would allow a more appropriate selection of the best device for each individual case. The main limitation is that this is a series of only two cases performed in a short interval of time, so further studies are needed to establish which devices are most appropriate in this type of patient, as well as the exact indication for endovascular treatment. To date, few clinical trials have been conducted to assess the success or failure of endovascular devices in CVT, so our work aims to provide new information for the future conduct of randomized clinical trials that provide higher quality scientific evidence in the treatment of this disease.

In conclusion, we present two cases of DCVT refractory to anticoagulation therapy and successfully treated by rescue MT with stent retriever devices, including the novel Tiger XL^®^. Anticoagulation is the first-line treatment in CVT. However, some situations predictive of poor functional prognosis such as DCVT with progressive neurological deterioration despite anticoagulation or the development of intracranial hemorrhage may require endovascular treatment with MT, with increasing evidence that it improves vital and functional prognosis.

## Data Availability

The original contributions presented in the study are included in the article/supplementary material, further inquiries can be directed to the corresponding author.
